# LINK-A lncRNA promotes migration and invasion of ovarian carcinoma cells by activating TGF-β pathway

**DOI:** 10.1042/BSR20180936

**Published:** 2018-09-14

**Authors:** Jiezhi Ma, Min Xue

**Affiliations:** Department of Obstetrics and Gynecology, Xiangya Third Hospital, Central South University, Changsha City, Hunan Province, 410013, P.R. China

**Keywords:** invasion, LINK-A lncRNA, migration, ovarian carcinoma, TGF-β1

## Abstract

**Introduction**: LINK-A lncRNA is a well-characterized oncogenic lncRNA only in triple negative breast cancer. Our study was carried out to investigate the possible involvement of LINK-A lncRNA in ovarian carcinoma. **Methods**: Expression of LINK-A in ovarian biopsies and plasma of both ovarian carcinoma patients and healthy females was detected by qRT-PCR. Plasma TGF-β1 was detected by ELISA. Correlation between plasma LINK-A and TGF-β1 was analyzed by Pearson correlation analysis. Correlation between plasma LINK-A and patients’ clinicopathological data was analyzed by Chi-square test. LINK-A overexpression vector was transfected into cells of human ovarian carcinoma cell lines. Cell migration and invasion were detected by Transwell migration and invasion assay. TGF-β1 expression was detected by Western blot. **Results:** We found that LINK-A and TGF-β1 were up-regulated in ovarian carcinoma patients than in healthy controls. Plasma levels of LINK-A were positively correlated with plasma TGF-β1 in ovarian carcinoma patients but not in healthy controls. Plasma levels of LINK-A were correlated with distant tumor metastasis but not tumor size. LINK-A overexpression led to up-regulated TGF-β1 in ovarian carcinoma cells and promoted cell migration and invasion. In contrast, TGF-β1 treatment showed no effects on LINK-A expression but attenuated the effects of LINK-A overexpression on cell migration and invasion. **Conclusions:** We conclude that LINK-A lncRNA may promote migration and invasion of ovarian carcinoma cells by activating TGF-β pathway.

## Introduction

Tumor metastasis is the main challenge for the treatment of variety types of human malignancies [1]. Ovarian carcinoma is one of the leading causes of cancer-related deaths in female residents in both developed and developing countries [2]. Most patients with ovarian carcinoma showed no obvious symptoms at early stages and tumor metastasis within the abdomen is frequently observed by the time of diagnosis [3]. Tumor metastasis as the most deadly aspect of cancers is achieved through cell proliferation, angiogenesis, cell adhesion, cell migration, and cell invasion into the surrounding tissues [4]. Prevention of tumor metastasis is considered to be a major task in clinical practices nowadays.

TGF-β signaling is a sword in cancer biology that inhibits tumor growth at early stages but promotes tumor metastasis at later stages through the activation of epithelial–mesenchymal transition (EMT) [5]. It is known that the invasion of carcinoma cells is at least partially mediated through the activation of TGF-β signaling [6]. TGF-β signaling achieves its biological roles in physiological processes and pathological changes by interacting with different signaling molecules including lncRNAs [7], which is a subgroup of non-coding RNAs composed of more than 200 nucleotides and plays critical roles in human diseases including different types of malignancies [8]. LINK-A lncRNA has been reported to play an oncogenic role in triple negative breast cancer (TNBC). In our study, we observed that LINK-A may promote the metastasis of ovarian carcinoma by up-regulating TGF-β1.

## Materials and methods

### Patients

From January 2015 to January 2018, a total of 148 patients with ovarian carcinoma were treated in Xiangya Third Hospital. Among those patients, 68 cases were enrolled in the present study according to inclusion and exclusion criteria. Inclusion criteria: (1) patients diagnosed as ovarian carcinoma through ovarian biopsies; (2) diagnosis and treatment were performed for the first time; (3) patients and their families were willing to participate. Exclusion criteria: (1) patients complicated with other malignancies; (2) patients complicated with other ovarian diseases; (3) patients who were treated before admission. Age of those patients ranged from 28 to 71 years, with a mean age of 50.1 ± 6.2 years. At the same time, a total of 34 healthy females were also enrolled to serve as control group. Age of control group ranged from 26 to 68 years, with a mean age of 48.4 ± 7.0 years. No significant differences in age, living habits including smoking and drinking, body mass index (BMI), and other basic clinical data were found between two groups. All participants signed informed consent. The ethics committee of Xiangya Third Hospital approved the present study.

### Specimen collection

All ovarian carcinoma patients received ovarian biopsies. Ovarian biopsies were also performed on those 34 healthy females to detect potential ovarian lesions, while those lesions were excluded after pathological examinations. Blood was extracted from elbow vein of both patients and healthy controls on the day of admission. Blood was kept in BD Vacutainer plasma preparation tubes at room temperature for 30 min, followed by centrifugation at 1200 ***g*** for 15 min to collect plasma. All specimens were stored in liquid nitrogen before use.

### Real-time quantitative PCR

Trizol reagent (Invitrogen, U.S.A.) was used to extract total RNA in strict accordance with manufacturer’s instruction. Total RNA samples with good quality were subjected to reverse transcription to obtain cDNA. cDNA was tested by routine PCR before qRT-PCR. SYBR^®^ Green Real-Time PCR Master Mixes (Thermo Fisher Scientific, U.S.A.) was used to prepare PCR reaction systems. Primers of lncRNA LINK-A were: 5′-TTCCCCCATTTTTCCTTTTC-3′ (upstream) and 5′-CTCTGGTTGGGTGACTGGTT-3′ (downstream) for human LINK-A; Primers for β-actin endogenous control were: 5′-GACCTCTATGCCAACACAGT-3′ (forward) and 5′-AGTACTTGCGCTCAGGAGGA-3′ (reverse). All primers were synthesized by Sangon (Shanghai, China). Parameters of PCR reactions were: 40 s at 95°C, then 40 cycles of 15 s at 95°C and 30 s at 57°C. Data normalization was performed using 2^−ΔΔ*C*^_T_ method.

### Enzyme-linked immunosorbent assay (ELISA)

ELISA for plasma TGF-β1 was completed according to manufacturer’s instructions (DY240, R&D Systems). TGF-β1 plasma levels were normalized for platelet activation by using PF4 ELISA.

### Cell culture and transfection

Two human ovarian carcinoma cell lines UWB1.289 (ATCC^®^ CRL-2945™) and UWB1.289+BRCA1 (ATCC^®^ CRL-2946™) were purchased from ATCC (U.S.A.). A human normal ovarian epithelial cell line SV40 was purchased from ABM (New York, U.S.A.). Cells of all three cell lines were cultured in 50% ATCC-formulated RPMI-1640 medium and 50% MEGM medium supplemented containing 3% of fetal bovine serum (FBS) at 37°C in a 5% CO_2_ incubator. LINK-A cDNA surrounded by ECOR I was obtained through PCR amplification. This DNA fragment was inserted into ECOR I linearized pIRSE2 vector (Clontech, Palo Alto, CA, U.S.A.) to make a LINK-A expression vector. Lipofectamine 2000 reagent (cat. no. 11668-019; Invitrogen, Thermo Fisher Scientific, Inc.) was used to transfect 10 nM vectors into 5 × 10^5^ cells. Cells without transfection were control cells. Cells transfected with empty vector were negative control cells. An overexpression rate above 200% was achieved before subsequent experiments.

### Transwell migration and invasion assay

After transfection and confirmation of overexpression, cells of three cells lines were collected to prepare cell suspensions with a final density of 3 × 10^4^ cells/ml. Transwell migration and invasion assay was performed to measure *in vitro* cell migration and invasion abilities. For migration assay, 3 × 10^3^ cells/0.1 ml of 1% FBS culture medium were transferred to the upper Transwell chamber, and the lower chamber was filled with 20% FBS RPMI-1640 medium (Thermo Fisher Scientific, U.S.A.). Migration was allowed for 2 h, and migrating cells were stained with 0.5% Crystal Violet (Sigma-Aldrich, U.S.A.) at room temperature for 15 min and were counted under an optical microscope. For invasion assay, upper chamber was coated with Matrigel (356234, Millipore, U.S.A.) before use and all other steps were the same. Cell migration and invasion were normalized to control cells.

### Western blot

Total protein was extracted from cells using RIPA solution (Thermo Fisher Scientific, U.S.A.) in strict accordance with manufacturer’s instructions. Protein concentration was measured by BCA assay, followed by 10% SDS-PAGE gel electrophoresis with 20 μg denatured protein in each well. After gel transfer, blocking was performed by incubating membranes with 5% skimmed milk at room temperature for 1 h. After washing with TBST (0.3% Tween), membranes were incubated with rabbit anti-human primary antibodies of TGF-β1 (1: 1200, ab9758, Abcam) and GAPDH primary antibody (1: 1400, ab8245, Abcam) overnight at 4°C. After washing with TBST (0.3% Tween), membranes were further incubated anti-rabbit IgG-HRP secondary antibody (1:1000, MBS435036, MyBioSource) for 4 h at room temperature. Signals were developed using ECL (Sigma-Aldrich, U.S.A.) and scanned using MYECL™ Imager (Thermo Fisher Scientific, U.S.A.). Data normalization was performed using Image J V 1.6 software.

### Statistical analysis

SPSS19.0 (SPSS Inc., U.S.A.) was used for all statistical analyses. Gene expression data and cell migration as well as invasion data were recorded as mean ± standard deviation and compared by either *t* test between two groups and one-way analysis of variance followed by LSD test among multiple groups. Chi-square test was performed to analyze correlations between LINK-A expression and patients’ clinicopathological data. *P*<0.05 was considered to be statistically significant.

## Results

### Expression of LINK-A was up-regulated in patients with ovarian carcinoma than in healthy females

**Figure 1:**
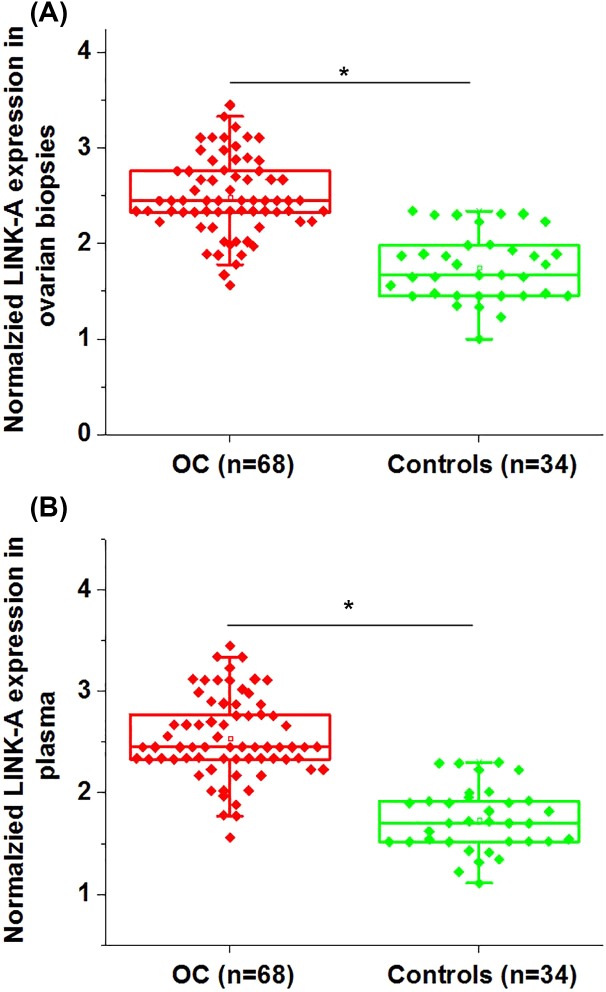
Expression of LINK-A was up-regulated in patients with ovarian carcinoma than in healthy females This figure shows the normalized expression levels of LINK-A in ovarian biopsies (**A**) and plasma (**B**) of patients with ovarian carcinoma and healthy females. Notes: *P<0.05; OC, ovarian carcinoma.

qRT-PCR was performed to investigate the expression of LINK-A in ovarian biopsies and plasma of 68 patients with ovarian carcinoma and 34 healthy females. As shown in Figure 1A, expression levels of LINK-A in ovarian biopsies were significantly higher in ovarian carcinoma patients than those in healthy females (*P*<0.05). In addition, plasma levels of LINK-A were also significantly higher in ovarian carcinoma patients than those in healthy females (*P*<0.05, Figure 1B).

### Plasma levels of TGF-β1 were higher in patients with ovarian carcinoma than in healthy females

**Figure 2:**
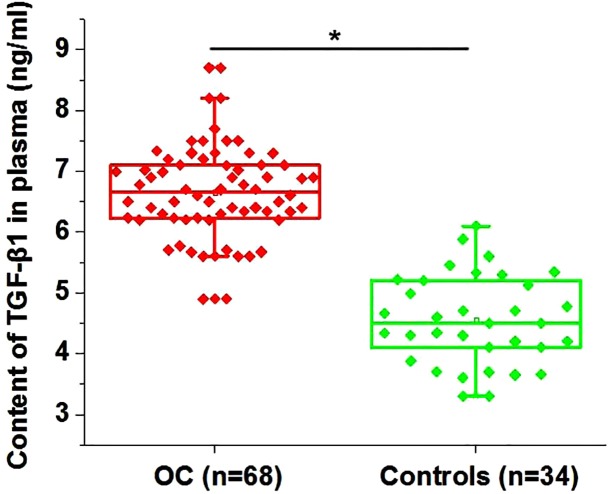
Plasma levels of TGF-β1 were lower in patients with ovarian carcinoma than in healthy females Notes:*P<0.05; OC, ovarian carcinoma.

ELSIA was performed to measure plasma levels of TGF-β1 in patients with ovarian carcinoma and healthy females. As shown in Figure 2, plasma levels of TGF-β1 were significantly higher in ovarian carcinoma patients than in healthy females (*P*<0.05).

### Plasma levels of LINK-A are positively correlated with plasma levels of TGF-β1 in ovarian carcinoma patients but not in healthy females

**Figure 3:**
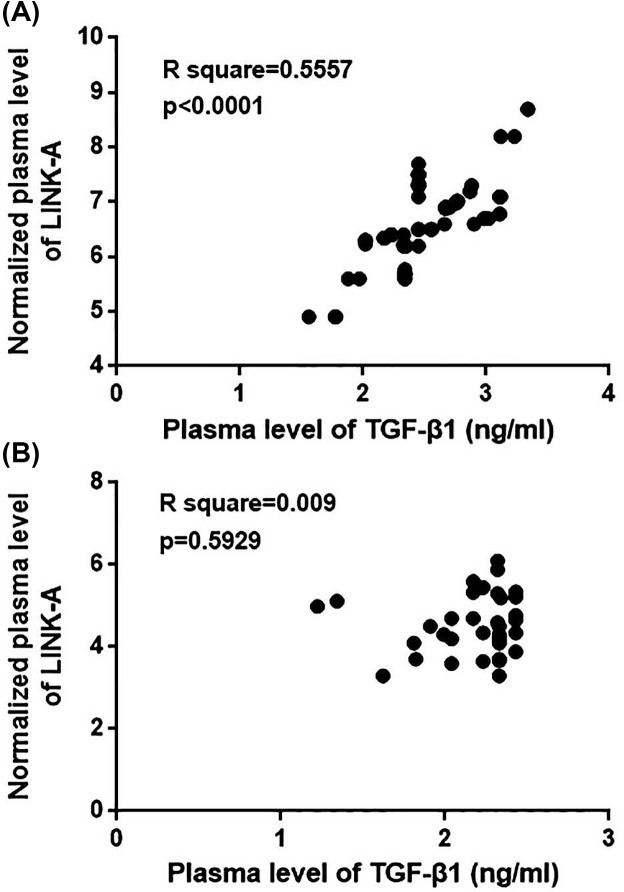
Plasma levels of LINK-A are positively correlated with plasma levels of TGF-β1 in ovarian carcinoma patients but not in healthy females This figure shows the Pearson correlation analysis of the correlation between plasma levels of LINK-A and plasma levels of TGF-β1 in ovarian carcinoma patients (**A**) and in healthy females (**B**).

Pearson correlation analysis was performed to investigate the correlation between plasma levels of LINK-A and TGF-β1 in ovarian carcinoma patients. As shown in Figure 3A, a significantly positive correlation was found between plasma levels of LINK-A and TGF-β1 in ovarian carcinoma patients (*P*<0.0001). In contrast, no significant correlation was found between plasma levels of LINK-A and TGF-β1 in healthy females (*P*>0.05, Figure 3B).

### Plasma levels of LINK-A were significantly correlated with tumor metastasis in ovarian carcinoma patients

Patients were divided into high- and low-expression group (*n*=34) according to the median plasma levels of LINK-A. Chi square test was performed to investigate the correlation between plasma levels of LINK-A and clinicopathological data of patients with ovarian carcinoma. As shown in Table 1, plasma levels of LINK-A were significantly correlated with tumor distant metastasis, but not tumor size, age, smoking and drinking habits, and BMI.

**Table 1:** Correlation between plasma levels of LINK-A and clinicopathological data of patients with ovarian carcinoma

Items	Groups	Cases	High expression	Low expression	χ²	*P* value
Age	>50 (years)	33	16	17	0.06	0.81
	<50 (years)	35	18	17		
Smoking	<18.5	15	7	8	0.58	0.75
	18.5–23.9	39	21	18		
	>23.9	14	6	8		
Smoking	Yes	18	8	10	0.30	0.58
	No	50	26	24		
Drinking	Yes	23	13	10	0.59	0.44
	No	45	21	24		
Primary tumor diameter	>2 cm	39	17	22	1.50	0.22
	<2 cm	29	17	12		
Distant tumor metastasis	Yes	32	22	10	8.50	0.00
	No	36	12	24		

### LINK-A overexpression led to promoted expression of TGF-β1 in ovarian carcinoma cells

**Figure 4:**
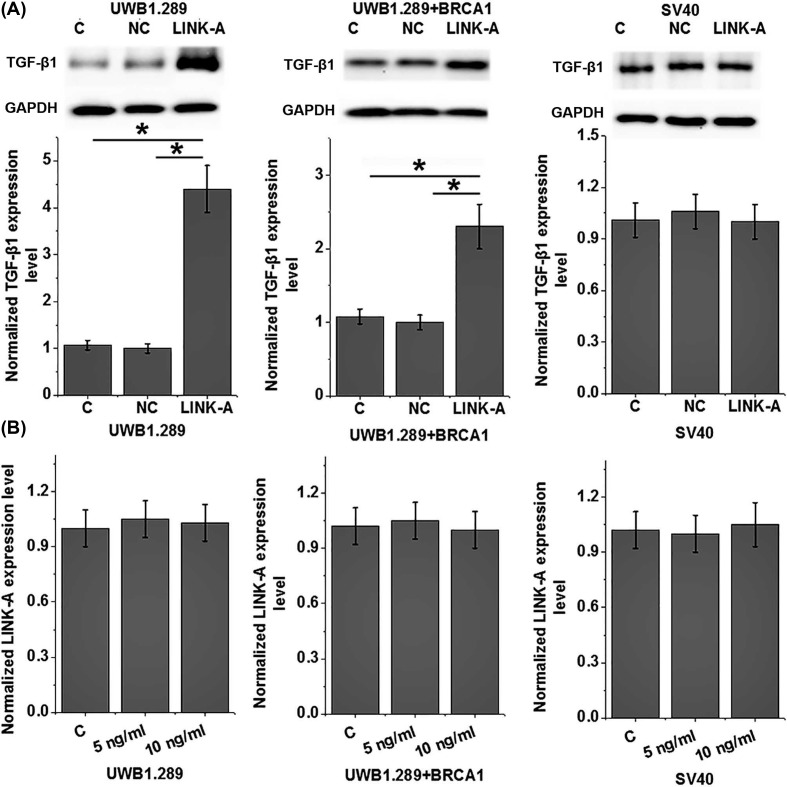
LINK-A overexpression promoted expression of TGF-β1 in ovarian carcinoma cells This figure shows the effects of LINK-A overexpression on (**A**) expression of TGF-β1 and (**B**) the effects of exogenous TGF-β1 treatment on LINK-A expression in cells of two human ovarian carcinoma cell lines UWB1.289 and UWB1.289+BRCA1 and a human normal ovarian epithelial cell line SV40; *P<0.05.

Our data suggest that LINK-A is likely involved in tumor metastasis. TGF-β1 signaling plays pivotal roles in different types of malignancies including ovarian cancer [11]. Our study also showed that expression level of LINK-A is positively correlated with expression level of TGF-β1 in ovarian carcinoma patients. Therefore, the potential interaction between LINK-A and TGF-β1 in ovarian carcinoma was explored by transfection of LINK-A expression vector into ovarian carcinoma cells. As shown in Figure 4A, LINK-A overexpression significantly promoted the expression of TGF-β1 in cells of two human ovarian carcinoma cell lines (*P*<0.05) but not in cells of the human normal ovarian epithelial cell line SV40 (*P*>0.05). In addition, treatment with exogenous TGF-β1 (Sigma-Aldrich) at doses of 5 and 10 ng/ml showed no significant effects on LINK-A in cells of all three cell lines (*P*>0.05, Figure 4B).

### LINK-A overexpression promoted migration and invasion of ovarian carcinoma cells

**Figure 5:**
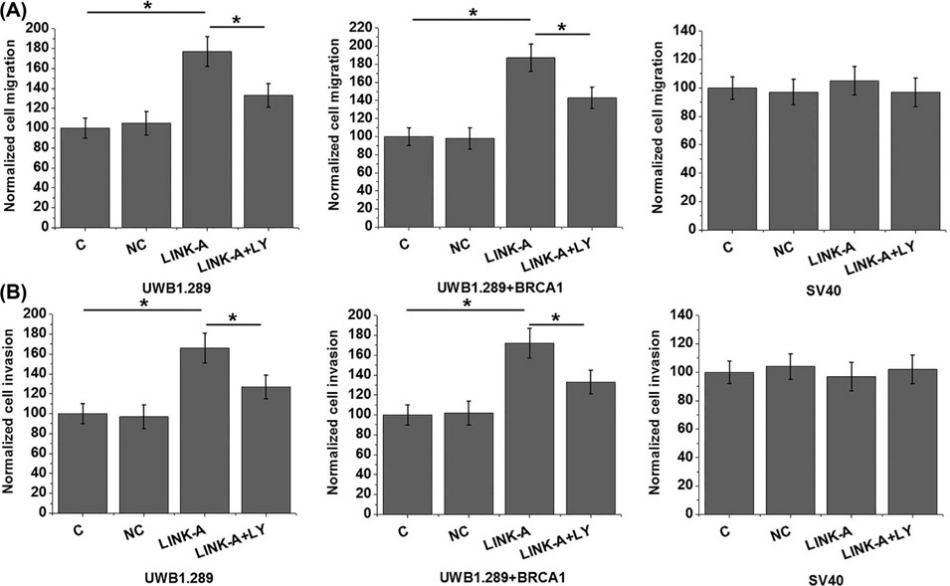
LINK-A overexpression promoted migration and invasion of ovarian carcinoma cells This figure shows the effects of LINK-A overexpression and treatment with TGF-β1 inhibitor on (**A**) migration and (**B**) invasion of cells of two human ovarian carcinoma cell lines and a human normal ovarian epithelial cell line SV40; *P<0.05.

Transwell migration and invasion assay was performed to investigate the effects of LINK-A overexpression on migration and invasion of ovarian carcinoma cells. As shown in Figure 5, LINK-A overexpression significantly promoted the migration (Figure 5A) and invasion (Figure 5B) of cells of two human ovarian carcinoma cell lines but not cells of the human normal ovarian epithelial cell line SV40. In addition, treatment with TGF-β inhibitor LY2109761 (LY, Sigma-Aldrich) at a dose of 100 nM significantly recued effect of LINK-A overexpression on cancer cell migration (Figure 5A) and invasion (Figure 5B).

## Discussion

LINK-A is a newly identified lncRNA with characterized function as an oncogenic role only in TNBC [9,10]. Our study first reported the oncogenic role of LINK-A in ovarian cancer. Our data also suggest that LINK-A may participate in the regulation of tumor metastasis in ovarian carcinoma through the up-regulation of TGF-β1 expression.

Differential expression in patients and healthy people indicates its involvement in the diseases [12]. Up-regulation of LINK-A has been reported in TNBC [9]. In our study, significant up-regulated expression of LINK-A in ovarian biopsies was found in ovarian carcinoma patients than in healthy controls. Therefore, up-regulation of LINK-A is likely involved in the pathogenesis of ovarian carcinoma. LINK-A is first identified as a cytoplasmic lncRNA [10]. In our study, LINK-A was detected in plasma of all ovarian carcinoma patients and healthy controls. Extracellular RNAs have been proved to serve as signaling molecule to systemically regulate body growth and development [13]. The existing of circulating LINK-A indicates its potential involvement in systemic regulations.

The onset and development of ovarian carcinoma are affected by various internal and external factors. It has been reported that alcohol intake [14] and frequent cigarette smoking [15] increase the risk of ovarian cancer. Most cases of ovarian cancers develop after menopause, and aging is a risk factor of this disease [16]. It also has been reported that overweight or obese females have significantly higher incidence of ovarian carcinoma compared with females with BMI in normal range [17]. In our study, plasma levels of LINK-A showed no significant correlations with patients’ age, BMI as well as smoking and drinking habits. Therefore, LINK-A may participate in the pathogenesis of ovarian carcinoma through pathways independent from those factors.

Our study also proved that plasma levels of circulating LINK-A are closely correlated with distant tumor metastasis but not tumor size. Therefore, LINK-A may only participate in tumor metastasis but not tumor growth in ovarian carcinoma. TGF-β signaling induces EMT to promote tumor metastasis [18]. In our study, significantly higher plasma levels of TGF-β1 were observed in ovarian carcinoma patients than in healthy controls. However, it is known that TGF-β can inhibit tumor growth at early stages [19]. The significantly up-regulated plasma levels of TGF-β1 in ovarian carcinoma patients than in healthy controls may be explained by the pathological stages of patients included in the present study. Our *in vitro* cell experiments suggest that TGF-β1 is likely an upstream regulator of LINK-A in the migration and invasion of ovarian carcinoma cells. However, a significant positive correlation between plasma levels of LINK-A and plasma levels of TGF-β1 was only observed in ovarian carcinoma patients but not in healthy controls. Those data suggest that the regulatory role of LINK-A on TGF-β1 expression is likely to be disease-specific. Our future study will focus on identification of the disease-related mediators between LINK-A and TGF-β1 in ovarian carcinoma.

It is also worth to note that LINK-A overexpression changed the biological behaviors of ovarian carcinoma cells but not normal ovarian cells. Therefore, LINK-A may serve as a potential therapeutic target for ovarian carcinoma.

## Conclusions

In conclusion, LINK-A is overexpressed in ovarian carcinoma. Overexpression of LINK-A may promote the metastasis of ovarian carcinoma by activating TGF-β signaling. However, our study only suggested a possible LINK-A–TGF-β1 sequential signaling in this disease. Disease-related mediators between LINK-A and TGF-β1 in ovarian carcinoma remain to be identified.

## Abbreviations

BMI: body mass index
EMT: epithelial–mesenchymal transition
TNBC: triple negative breast cancer
